# *CYP4F2* and *VKORC1* Polymorphisms Amplify the Risk of Carotid Plaque Formation

**DOI:** 10.3390/genes11070822

**Published:** 2020-07-20

**Authors:** Stefan Cristian Vesa, Sonia Irina Vlaicu, Vitalie Vacaras, Sorin Crisan, Octavia Sabin, Sergiu Pasca, Adrian Pavel Trifa, Tamas Rusz-Fogarasi, Madalina Sava, Anca Dana Buzoianu

**Affiliations:** 1 Department of Pharmacology, Toxicology and Clinical Pharmacology, “Iuliu Hațieganu” University of Medicine and Pharmacy, 400337 Cluj-Napoca, Romania; stefanvesa@gmail.com (S.C.V.); octaviasabin@gmail.com (O.S.); ancabuzoianu@yahoo.com (A.D.B.); 2 Department of Internal Medicine, 1st Medical Clinic, “Iuliu Haţieganu” University of Medicine and Pharmacy, 400006 Cluj-Napoca, Romania; vlaicus@yahoo.com; 3 Department of Neurosciences, “Iuliu Hațieganu” University of Medicine and Pharmacy, 400486 Cluj-Napoca, Romania; 4 Department of Internal Medicine, 5th Medical Clinic, “Iuliu Haţieganu” University of Medicine and Pharmacy, 400139 Cluj-Napoca, Romania; crisan.sorin@gmail.com; 5 Medfuture Research Center for Advanced Medicine, “Iuliu Hațieganu” University of Medicine and Pharmacy, 400349 Cluj-Napoca, Romania; 6 Department of Medical Genetics, “Iuliu Haţieganu” University of Medicine and Pharmacy, 400349 Cluj-Napoca, Romania; trifa.adrian@gmail.com; 7 Department of Surgery, 1st Surgical Clinic, Emergency County Hospital, 400006 Cluj-Napoca, Romania; rusztamas@yahoo.com; 8 Department of Dermatology, Emergency County Hospital, 410032 Oradea, Romania; madalina.sava0508@gmail.com

**Keywords:** atherosclerosis, *VKORC1* polymorphism, *CYP4F2* polymorphism

## Abstract

Introduction: Atherosclerosis represents the process by which fibrous plaques are formed in the arterial wall, increasing its rigidity with a subsequent decrease in blood flow which can lead to several cardiovascular events. Seeing as vitamin K antagonists are involved in the pathogenesis of atherosclerosis, we decided to investigate whether polymorphisms in genes that influence vitamin K metabolism might have an impact in modulating the risk of plaque formation. Patients and Methods: In the current study we included adult patients admitted in the Clinical Municipal Hospital of Cluj-Napoca without any carotid or femoral plaques clinically visible at the initial investigation, and a five year follow-up was subsequently performed. We recorded the following patient characteristics: age at inclusion, gender, area of living, smoking, presence of carotid and/or femoral plaques at five years, ischemic heart disease, arterial hypertension, atrial fibrillation, heart failure, diabetes mellitus, obesity, dyslipidemia, drug (oral anticoagulants, antihypertensives, hypolipidemic, anti-diabetic) use and status for the following gene polymorphisms: VKORC1 1639 G>A, CYP4F2 1347 G>T and GGCX 12970 C>G. Results: We observed that the major predictor of both carotid and femoral plaque formation is represented by ischemic cardiac disease. VKORC1 and CYP4F2 polymorphisms did not predict plaque formation, except for VKORC1 homozygous mutants. Nonetheless, both VKORC1 and CYP4F2 interacted with ischemic cardiac disease, increasing the risk of developing a carotid plaque, while only CYP4F2, but not VKORC1, interacted with ischemic cardiac disease to increase the risk of femoral plaque formation. Conclusions: We documented that CYP4F2 and VKORC1 polymorphisms boost the proinflammatory plaque environment (observed indirectly through the presence of ischemic heart disease), increasing the risk of plaque development.

## 1. Introduction

Atherosclerosis represents the process by which fibrous plaques are formed in the arterial wall with a subsequent increase in its rigidity, diminished blood flow, with an increase in systolic blood pressure, leading to critical cardiovascular events such as stroke and myocardial infarction [1]. Several reports have illustrated that vitamin K as well as vitamin K antagonists have an important impact in the pathogenic processes that occur during the atherosclerotic plaque formation [2].

Specifically, it has been shown that vitamin K has an anti-inflammatory effect through the inhibition of the NFκβ pathway and macrophage M1 polarization—key players in the formation of atherosclerotic plaques [3]. Thus, one can infer that vitamin K antagonists favor a pro-inflammatory state triggering an accelerated plaque evolution.

Plaque calcification represents one of the later processes that occur in the evolution of the atherosclerotic plaque, changing its stability, decreasing lumen permeability, and stiffening the vascular wall [4]. One of the factors that influence calcium metabolism is represented by vitamin K [5], involved in γ carboxylated protein formation [6,7]. Because these γ carboxylated residues possess two adjacent negative charges, they can chelate calcium ions, leading to vitamin K reduction at the site of interest. One such protein is Matrix Gla protein (MGP), which inhibits plaque calcification both in a direct and an indirect manner. The direct manner is consisted in the chelation of calcium ions, while MGP indirectly inhibits bone morphogenetic proteins that stimulate the formation of osteoblast-like cells and the initiation of the calcification process [8]. Moreover, the role of MGP has also been validated in a murine double knock-out model, in which MGP deficiency was directed to early calcification of their growth cartilages; furthermore, the same phenotype was reproduced by means of a diet containing warfarin and phylloquinone [9].

The activity of VKORC1 [10,11,12,13] and CYP4F2 [14,15,16,17,18] are able to influence the metabolism and implicitly the active levels of vitamin K and its antagonists. Moreover, vitamin K acts as a cofactor for GGCX [19,20,21,22,23] in the process of generating γ carboxylated proteins. Thus the polymorphisms influencing the activity of these enzymes can impact vitamin K metabolism, influence atherosclerotic plaque formation and increase the risk of the occurrence of cardiovascular events as previously observed in the case of *VKORC1* polymorphisms [12].

In light of the above information, in the current study, we aimed to assess the influence of polymorphisms in the *VKORC1*, *CYP4F2*, and *GGCX* genes on the formation of atherosclerotic plaques in patients with various cardiovascular diseases (arterial hypertension, atrial fibrillation, ischemic heart disease).

## 2. Patients and Methods

This was a prospective, observational, analytical, cohort study.

In the current study, we included adult patients without any carotid or femoral plaques clinically visible at the initial investigation and for whom a five-year follow-up was subsequently performed. This study was performed in accordance with the Declaration of Helsinki and was approved by the Ethics Committee of “Iuliu Hațieganu” University of Medicine and Pharmacy, Cluj-Napoca, Romania (no. 350/13.11.2014).

The patients were included from those admitted for various cardiovascular diseases (arterial hypertension, atrial fibrillation, ischemic heart disease) at the Clinical Municipal Hospital of Cluj-Napoca in the timeframe January 2010–December 2011.

We did not include patients with a history of acute coronary syndrome, heart failure with left ventricle ejection fraction under 50%, stroke, cancer, autoimmune diseases, or liver cirrhosis.

The following data were recorded for each patient: age, gender, living area, smoking status, presence of ischemic heart disease, atrial fibrillation, arterial hypertension, heart failure, diabetes mellitus, obesity (BMI > 30 kg/m^2^), dyslipidemia, and drug (oral anticoagulants, antihypertensives, hypolipidemic, anti-diabetic) use. We recorded the drug use only in patients that followed the same treatment for all 5 years.

Peripheral blood was collected in a vacutainer containing EDTA (ethylenediamine tetraacetic acid). DNA extraction was performed using a genomic DNA purification kit (Wizard Genomic DNA Purification Kit, Promega, Madison, WI, USA). Genotyping of the *VKORC1* [24,25,26], *CYP4F2* [27], and *GGCX* [27] polymorphisms was performed as previously described. *VKORC1* and *CYP4F2* mutational status was dichotomized between no mutation and either monoallelic or biallelic mutation.

Ultrasound examination was performed by the same experienced physician on all patients before inclusion in the study and after five years using an Aloka SSD 4000 unit with a linear transducer at a frequency between 7 and 10 MHz. The physician had a 15 years’ experience in vascular ultrasonography and performed more the 10,000 examinations at the moment of the study. The examination was performed on carotid arteries on both sides (common, internal, and external) and on common and superficial femoral arteries on both lower limbs. The presence of carotid/femoral plaques was noted according to the following criteria: localized protrusion of the carotid wall, which was thicker than 1.5 mm, or more than 50% of the intima-media thickness of the adjacent area [28].

Statistical analysis was performed using R 4.0.1 (R Foundation for Statistical Computing, Vienna, Austria). Categorial variables were presented as absolute and relative value. Contingency tables were analyzed using Fisher’s test. Normality of the distribution was assessed using the Shapiro–Wilk test and histogram visualization. Non-normally distributed variables were represented as median (quartile 1, quartile 3). Univariate logistic regression was used to determine the association of other variables with a dependent variable. Variables that presented a *p* value of < 0.1 in the univariate logistic regression were used for the multivariate logistic regression. The adjusted association of variables with the selected endpoints was assessed using multivariate logistic regression. Interactions between two variables were assessed using logistic regression. A *p* value under 0.05 was considered statistically significant.

## 3. Results

Our initial cohort included 270 patients. At the five years follow-up only 76 patients remained in the final group. Cohort descriptive statistics are presented in Table 1. Information about the relevant medication is presented in Supplementary Table S1.

The first strategy we undertook was to determine which were the predictors of plaque formation in our cohort in the univariate (Table 2) and multivariate logistic regression model (Table 3). It has to be mentioned that although insulin presented a *p* value under 0.1 regarding its association with carotid plaque formation, there were only 6 patients taking insulin. Because of this, we considered insulin use not to be appropriate for the multivariate model.

We observed that ischemic heart disease and obesity were the variables that predicted the formation of carotid plaques and of any of the two plaques, but not of femoral plaques alone. We ought to mention that when using *VKORC1* homozygous mutant as the event, we found a statistically significant association with carotid plaque formation (OR = 3.857, 95%CI 0.978–14.721, *p* = 0.0469). When assessing the relationship between carotid plaque formation and *VKORC1* without dichotomizing it, we observed a tendency for statistical significance (*p* = 0.0847). Furthermore, we observed that there was a statistically significant association between femoral and carotid plaque formation (*p* = 0.022).

After this we decided to assess whether an interaction (Table 4) exits between mutations in *VKORC1* and *CYP4F2* and the variables that were statistically significant in the univariate and multivariate logistic models.

**Figure 1:**
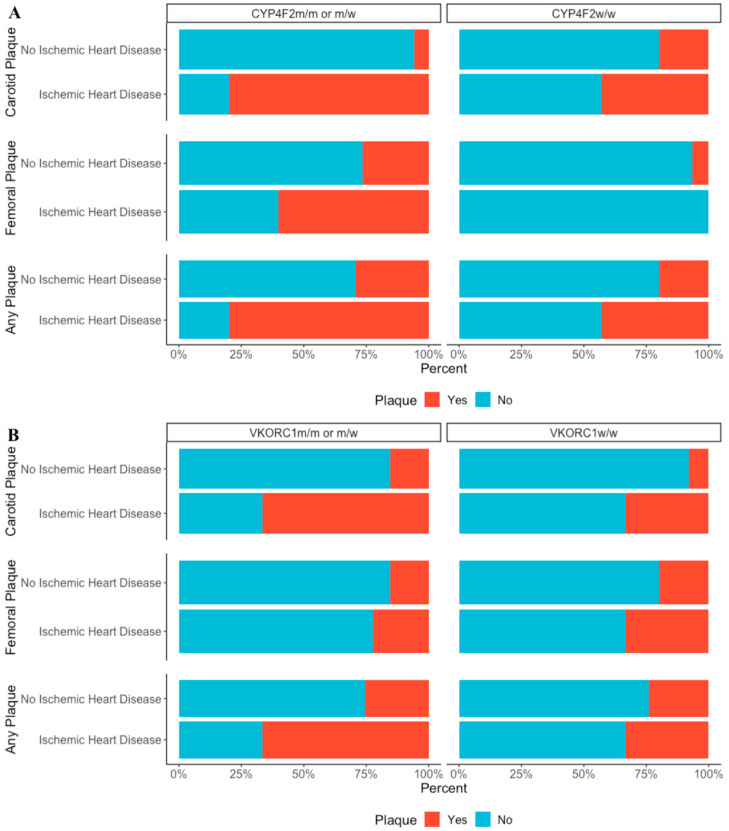
(**A**) *CYP4F2* interaction with ischemic heart disease in predicting plaque formation. (**B**) *VKORC1* interaction with ischemic heart disease in predicting plaque formation.

We observed that *CYP4F2* interacted with the presence of ischemic heart disease increasing the risk of plaque development in the carotid artery, femoral artery, or any of the two. Of note, we acknowledge that when assessing all interactions two by two, this was the only statistically significant interaction that was present in all three conditions (Figure 1A).

We further observed that *VKORC1* interacted with the presence of ischemic heart disease only in the case of carotid or any of the two plaques (Figure 1B). As obesity was also statistically significant in the previous model, we also assessed its interaction with mutations in the selected genes and noted that obesity interacted with *CYP4F2* in the formation of femoral plaques and with either the presence of *CYP4F2* or *VKORC1* in the development of either carotid or femoral plaques. Moreover, considering the known effect of these enzymes on the vitamin K antagonist metabolism, we wanted to establish whether there were interactions between polymorphisms in *VKORC* and *CYP4F2* and anticoagulant use. Interestingly, we found that anticoagulant use interacted only with *CYP4F2* in determining the formation of femoral plaque.

## 4. Discussion

Our data exposes ischemic heart disease as the factor with the most important predictive value regarding the formation of carotid or femoral plaques. Interestingly, *VKORC1* and *CYP4F2* polymorphisms did not predict plaque formation when considering both homozygous and heterozygous mutations as risk events. Herewith, these polymorphisms have an important association with ischemic heart disease and increased the risk of plaque development. Nonetheless, we also found that, when comparing *VKORC1* homozygous mutant patients with the others, there was a tendency for statistical significance for homozygous mutant patients to have a higher risk of plaque development. When separating *VKORC1* in homozygous mutant, homozygous wild type and heterozygous, we also observed a tendency for an escalating trend for the risk of plaque development with the more alleles of *VKORC1* mutated. This is in accordance with the activity influencing effect that the selected polymorphism is known to have [29]. Considering this and the fact that previous work has proven the role of *VKORC1* in influencing plaque formation [30], it is possible that *VKORC1* polymorphism might have a significant impact on plaque formation in a larger longitudinal study.

Other studies have certified that ischemic heart disease and atherosclerosis are frequently associated, through a possible common etiology consisting of both environmental and genetic factors that predispose to plaque development [31]. Data reported by us—that ischemic heart disease has an important effect on increasing the risk of developing plaques—it is likely that patients that have developed ischemic heart disease already have a significant proinflammatory phenotype (hypothesis already tested), which would recommend inflammation as an ideal target for the treatment of ischemic heart disease [32].

Moreover, others have determined that vitamin K antagonists accelerate plaque formation [33] and increase the calcification of atherosclerotic plaques [33]. Since vitamin K antagonists determine lower levels of vitamin K, one can infer that polymorphisms that have a similar effect would also have an impact in the formation of atherosclerotic plaques; this is harmony with studies demonstrating *VKORC1* mutations are associated with cardiovascular events [8]. In light of this, our findings emphasizing the effect of *VKORC1* and *CYP4F2* on increasing the risk of plaque development once ischemic heart disease is already present are noteworthy. Combining this piece of evidence with the fact that heart disease implies an underlying inflammatory phenotype [32] and that vitamin K has an anti-inflammatory role on macrophages [3] we hypothesize that one of the mechanisms through which *VKORC1* and *CYP4F2* polymorphisms interact with ischemic heart disease is by enhancing an already existing inflammatory environment.

Additionally, we observed curious differences between the variables that interact for the prediction of carotid and femoral plaques. Previous groups have described that femoral plaques are more frequently fibrocalcific, while carotid plaques more frequently present fibrous caps. Additionally, osteoid metaplasia is found more often in femoral plaques that in carotid plaques [34]. Of note, femoral plaques also contain a higher content of calcium and lower content of cholesterol compared to carotid plaques [34].

This is probably why anticoagulant use interacted with *CYP4F2* for the formation of femoral plaques, but not that of carotid plaques, as the influence of vitamin K antagonists in calcium metabolism is well established [5], although inflammatory mechanisms have also been described [3]. The interaction between the *VKORC* and *CYP4F2* polymorphisms and ischemic heart disease is more relevant for the prediction of carotid artery plaque formation than that of femoral artery plaques. We contemplate the possibility that carotid plaques form in a more inflammatory environment, while femoral plaques require a more important presence of excessive calcium at the situs of plaque formation. This hypothesis will be verified in future studies from our team.

The limitation of this study is mainly reflected by the low number of patients included, due to the requirement for them to have follow up at 5 years, which meant that some of those initially included were later excluded (drop-out). A potential bias that might have occurred following this is the fact that some of the patients that did not complete the 5 year follow up might have died of a plaque-related event. Nonetheless, this limitation of our study is in direct relationship with its main strength, as our results stand as the first longitudinal study to assess the effect that *VKORC1* and *CYP4F2* polymorphisms have on boosting the risk of plaque development at 5 years. Until now, other studies have determined the role of this polymorphisms in a cross-sectional manner [12,30,35]. Therefore, the current study significantly advances the knowledge in the field of atherosclerotic plaque formation.

## 5. Conclusions

Thus, we have documented that *CYP4F2* and *VKORC1* polymorphisms boost the proinflammatory plaque environment (observed indirectly through the presence of ischemic heart disease), increasing the risk of plaque development. Moreover, we describe different interactions that contribute to the prediction of carotid or femoral plaques formation. The observed difference might have un underlining explanation in the distinctive composition of the two plaque topographies.

As future research objectives, we aim to validate our findings with a larger cohort and to better underline the differences between carotid and femoral plaques.

## Figures and Tables

**Table 1:** Cohort characteristics.

Variable		Value (Percent)
Age (years)		68 (55, 74)
Age	≥65	41 (53.95%)
	<65	35 (46.05%)
Gender	Male	36 (47.37%)
	Female	40 (52.63%)
Area	Urban	43 (56.58%)
	Rural	33 (43.42%)
Ischemic heart disease	Yes	12 (15.79%)
	No	64 (84.21%)
Arterial hypertension	Yes	51 (67.11%)
	No	25 (32.89%)
Atrial fibrillation	Yes	16 (21.05%)
	No	60 (78.95%)
Heart failure	Yes	19 (25.00%)
	No	57 (75.00%)
Diabetes mellitus	Yes	23 (30.26%)
	No	53 (69.74%)
Obesity	Yes	24 (31.58%)
	No	52 (68.42%)
Dyslipidemia	Yes	61 (80.26%)
	No	15 (19.74%)
Anticoagulant use	Yes	59 (77.63%)
	No	17 (22.37%)
*VKORC1* (G-1639A) polymorphism	*w*/*w*	28 (36.84%)
	*m*/*w*	36 (47.37%)
	*m*/*m*	12 (15.79%)
*CYP4F2* (1347 C>T) polymorphism	*w*/*w*	37 (48.68%)
	*m*/*w*	33 (43.42%)
	*m*/*m*	6 (7.89%)
*GGCX* (12970 C>G) polymorphism	*w*/*w*	67 (88.16%)
	*m*/*w*	9 (11.84%)
	*m*/*m*	0 (0.00%)
Carotidian plaque at 5 years	Yes	15 (19.74%)
	No	61 (80.26%)
Femoral plaque at 5 years	Yes	14 (18.42%)
	No	62 (81.58%)
Smoking	Yes	12 (15.79%)
	No	64 (84.21%)

**Table 2:** Univariate logistic regression model for predicting carotid, femoral, or any of the two plaques. *VKORC1* and *CYP4F2* mutational status was dichotomized between no mutation and either monoallelic or biallelic mutation. *p* values were bolded if *p* < 0.05. ACE = angiotensin-converting enzyme. ARB = angiotensin II receptor blocker.

Variable	Carotidian Plaque				Femoral Plaque				Any Plaque			
	OR	95% Lower CI	95% Upper CI	*p*	OR	95% Lower CI	95% Upper CI	*p*	OR	95% Lower CI	95% Upper CI	*p*
Age ≥ 65 years	0.695	0.218	2.171	0.529	0.579	0.172	1.860	0.359	0.546	0.199	1.460	0.23
Male gender	0.689	0.208	2.147	0.525	1.138	0.35	3.700	0.827	0.799	0.293	2.133	0.655
Urban area	0.849	0.271	2.705	0.778	1.029	0.32	3.458	0.962	0.997	0.372	2.719	0.995
Ischemic heart disease	9.800	2.563	41.082	**0.00106**	1.606	0.319	6.461	0.525	4.200	1.182	16.025	**0.0279**
Arterial hypertension	1.444	0.433	5.711	0.568	2.02	0.558	9.610	0.319	1.583	0.552	5.000	0.407
Atrial fibrillation	0.516	0.075	2.178	0.419	1.028	0.21	3.898	0.97	0.719	0.182	2.380	0.607
Heart failure	0.398	0.058	1.647	0.256	0.784	0.162	2.903	0.733	0.533	0.137	1.711	0.318
Diabetes mellitus	0.804	0.202	2.699	0.735	0.905	0.226	3.094	0.879	1.012	0.335	2.880	0.983
Obesity	3.214	1.003	10.607	**0.0493**	2.647	0.798	8.870	0.108	3.727	1.329	10.819	**0.0132**
Dyslipidemia	0.605	0.169	2.496	0.455	3.792	0.662	71.862	0.218	1.244	0.371	4.942	0.735
Anticoagulant use	0.49	0.144	1.810	0.261	4.522	0.799	85.385	0.161	0.742	0.241	2.445	0.609
*VKORC1* (G-1639A) polymorphism	2.778	0.785	13.111	0.142	0.733	0.226	2.480	0.606	1.500	0.54	4.470	0.447
*CYP4F2* (1347 C>T) polymorphism	0.566	0.171	1.762	0.331	7.778	1.914	52.748	**0.0109**	1.742	0.651	4.849	0.275
*GGCX* (12970 C>G) polymorphism	0.473	0.024	2.905	0.497	NA	NA	NA	NA	0.256	0.013	1.521	0.212
β blocker	1.023	0.255	3.488	0.973	1.150	0.284	3.992	0.832	0.983	0.305	2.916	0.976
ACE inhibitor or ARB	1.181	0.378	3.759	0.773	1.000	0.308	3.248	1.000	0.883	0.328	2.359	0.803
Calcium channel blocker	2.462	0.714	8.172	0.142	1.905	0.516	6.503	0.310	2.036	0.675	6.044	0.199
Thiazide diuretic	0.952	0.268	3.059	0.936	1.085	0.301	3.565	0.896	1.037	0.359	2.869	0.945
Other antihypertensive medication	2.192	0.283	12.554	0.393	0.877	0.0438	6.075	0.908	1.167	0.153	6.467	0.864
Hypolipemiant medication	1.102	0.346	3.448	0.867	0.911	0.271	2.928	0.876	1.196	0.444	3.211	0.721
Insulin	4.833	0.811	29.047	0.072	0.877	0.044	6.075	0.908	2.500	0.431	14.526	0.286
Oral antidiabetic medication	1.653	0.401	5.926	0.455	1.136	0.231	4.355	0.860	1.725	0.511	5.553	0.363
Smoking	2.409	0.564	9.200	0.207	1.606	0.319	6.461	0.525	2.765	0.770	10.017	0.114

**Table 3:** Multivariate logistic regression for the significant variables that were selected from the univariate logistic regression model. *p* values were bolded if *p* < 0.05.

Variable	Carotidian Plaque				Any Plaque			
	OR	95% Lower CI	95% Upper CI	*p*	OR	95% Lower CI	95% Upper CI	*p*
Ischemic heart disease	11.883	2.847	58.525	**0.00106**	4.749	1.246	19.740	**0.0245**
Obesity	4.114	1.109	17.347	**0.0391**	4.076	1.388	12.654	**0.0119**

**Table 4:** Interaction assessment for the variables of interest. *VKORC1* and *CYP4F2* mutational status was dichotomized between no mutation and either monoallelic or biallelic mutation.

Variable 1	Variable 2	Carotidian Plaque				Femoral Plaque				Any Plaque			
		OR	95% Lower CI	95% Upper CI	*p*	OR	95% Lower CI	95% Upper CI	*p*	OR	95% Lower CI	95% Upper CI	*p*
Ischemic heart disease	*CYP4F2*	21.818	2.901	449.243	**0.00815**	8.181	1.225	67.878	**0.0302**	10.947	1.504	221.534	**0.0374**
Ischemic heart disease	*VKORC1*	12.889	2.889	70.509	**0.00126**	1.210	0.180	6.274	0.755	5.882	1.395	30.368	**0.0199**
Ischemic heart disease	*CYP4F2* or *VKORC1*	16.917	3.904	92.243	**0.000323**	2.143	0.414	9.123	0.319	7.292	1.804	37.034	**0.00786**
Femoral plaque	*CYP4F2*	2.409	0.564	9.200	0.207	NA	NA	NA	NA	NA	NA	NA	NA
Femoral plaque	*VKORC1*	5.182	1.083	25.148	**0.0349**	NA	NA	NA	NA	NA	NA	NA	NA
Femoral plaque	*CYP4F2* or *VKORC1*	3.313	0.857	12.217	0.072	NA	NA	NA	NA	NA	NA	NA	NA
Obesity	*CYP4F2*	2.101	0.499	7.818	0.279	5.893	1.561	22.693	**0.00838**	5.120	1.489	19.280	**0.011**
Obesity	*VKORC1*	2.550	0.678	8.971	0.148	1.136	0.231	4.355	0.860	2.461	0.754	7.979	0.129
Obesity	*CYP4F2* or *VKORC1*	3.231	0.973	10.736	0.052	2.571	0.739	8.701	0.127	4.481	1.529	13.719	**0.00688**
Anticoagulant use	*CYP4F2*	0.589	0.167	1.867	0.382	6.667	1.855	31.838	**0.00699**	1.662	0.620	4.518	0.312
Anticoagulant use	*VKORC1*	0.966	0.303	3.016	0.952	1.138	0.350	3.700	0.827	0.799	0.293	2.133	0.655
Anticoagulant use	*CYP4F2* or *VKORC1*	0.846	0.268	2.817	0.777	4.333	1.063	29.387	0.069	1.500	0.540	4.470	0.447

## Supplementary Materials

The following are available online at https://www.mdpi.com/2073-4425/11/7/822/s1, Table S1: Cohort characteristics regarding the relevant medication.
