# Supplementary material for: CYP4F2 and VKORC1 Polymorphisms Amplify the Risk of Carotid Plaque Formation
Source: Genes (Basel). 2020 Jul 20;11(7):822. doi: 10.3390/genes11070822 (PMC7396977; doi:10.3390/genes11070822)
Supplement: Supplementary file 1 [file genes-11-00822-s001.pdf]

Medication		Value (Percent)
Antihypertensive medication	Beta blocker	20 (26.32%)
	ACE inhibitor or ARB	38 (50.00%)
	Calcium channel blocker	19 (25.00%)
	Thiazide diuretic	26 (34.21%)
	Other	6 (7.89%)
Hypolipemiant medication		34 (44.74%)
Diabetes medication	Insulin	6 (7.89%)
	Oral antidiabetics	15 (19.74%)
